# The potential benefits of low-molecular-weight heparins in cancer patients

**DOI:** 10.1186/1756-8722-3-3

**Published:** 2010-01-14

**Authors:** Francisco Robert

**Affiliations:** 1 Department of Medicine, Division of Hematology/Oncology, Comprehensive Cancer Center, University of Alabama at Birmingham, 1802 6th Avenue South, NP CC-2555, Birmingham, AL 352943300, USA

## Abstract

Cancer patients are at increased risk of venous thromboembolism due to a range of factors directly related to their disease and its treatment. Given the high incidence of post-surgical venous thromboembolism in cancer patients and the poor outcomes associated with its development, thromboprophylaxis is warranted. A number of evidence-based guidelines delineate anticoagulation regimens for venous thromboembolism treatment, primary and secondary prophylaxis, and long-term anticoagulation in cancer patients. However, many give equal weight to several different drugs and do not make specific recommendations regarding duration of therapy. In terms of their efficacy and safety profiles, practicality of use, and cost-effectiveness the low-molecular-weight heparins are at least comparable to, and offer several advantages over, other available antithrombotics in cancer patients. In addition, data are emerging that the antithrombotics, and particularly low-molecular-weight heparins, may exert an antitumor effect which could contribute to improved survival in cancer patients when given for long-term prophylaxis. Such findings reinforce the importance of thromboprophylaxis with low-molecular-weight heparin in cancer patients.

## Introduction

Venous thromboembolism (VTE), comprising deep vein thrombosis (DVT) and pulmonary embolism (PE), is one of the principal causes of morbidity and mortality in surgical patients [1]. The development of post-surgical VTE is associated with significantly higher rates of hospital readmission, VTE recurrence, and a greater than 3-fold increase in mortality [2]. Cancer patients are at additional risk of VTE [3], and following cancer surgery VTE is the most common cause of death at 30 days [4].

The probability of diagnosing concomitant cancer is up to 10-times greater for cases of idiopathic VTE compared with those where the pre-disposing risk factor is known [5,6]. In a recent systematic review, the reported 12-month prevalence for cancer following VTE was 10.0% (95% confidence interval [CI]: 8.6-11.3) in patients with idiopathic VTE compared with 2.6% (95% CI: 1.6-3.6) in those with a provoked VTE [7]. Overall, around 10-20% of all non-cancer patients who present with idiopathic VTE develop cancer over the following 3 years [8]. The association between VTE and cancer is so pronounced that some researchers have argued that patients with idiopathic VTE be screened for occult cancer [7].

The risk of VTE in cancer patients varies according to disease-specific factors such as the location, stage, and type of the malignancy [3,9]. In addition, cancer patients typically present with a number of co-morbid conditions that predispose individuals to thrombosis, such as older age and frequent hospitalization [10,11]. Cancer patients undergoing surgery have up to twice the risk of DVT and three-times the risk of PE as non-cancer patients undergoing similar operations [12,13]. VTE risk is further increased by cancer therapies, with significant increases in VTE in cancer patients treated with chemo- and hormonal-therapy [11,14,15]. There is, therefore, a clear need for thromboprophylaxis in surgical cancer patients which is supported by current guidelines [1,16-19]. This review summarizes the relative merits of the low-molecular-weight heparins (LMWHs) and the other principal anticoagulants used for thromboprophylaxis in this high-risk population.

### Pathophysiology of VTE and cancer

Although the relationship between cancer and VTE was first recognized almost 150 years ago [20], the molecular basis of this association has only recently been investigated. Studies have shown a complex pathophysiology involving perturbation of multiple components within the coagulation and fibrinolytic pathways. The association between cancer and VTE works both ways, with cancer inducing a hypercoagulable state and the pro-thrombotic changes in turn facilitating cancer growth and metastasis [21]. Cancer cells have been shown to aberrantly express several components involved in coagulation. For example, tissue factor, a key activator of the coagulation cascade, is expressed on endothelial cells, monocytes and, most importantly, on tumor cells themselves and is thought to play a pivotal role in cancer-induced hypercoagulability [21]. In addition, cancerous cells can produce a cysteine proteinase, termed cancer procoagulant, which directly cleaves factor X to Xa leading to the generation of thrombin and thrombus formation [22]. von Willebrand factor (vWF) promotes platelet adhesion during thrombus formation and elevated vWF levels have been detected in various cancers [23]. Aberrant expression of glycoprotein IIb/IIIa receptors, which are involved in platelet activation and adhesion, and serve to promote and stabilize thrombi, is also observed on tumor cells [24].

Cancer is also associated with disturbances of the fibrinolytic system. Plasmin, which breaks down fibrin clots, is produced from its precursor molecule plasminogen in response to plasminogen activator or urokinase-type plasminogen activator, and is inhibited by plasminogen activator inhibitor [25]. However, deregulation of these factors is observed in cancer patients, resulting in disruptions to the normal process of clot lysis [25]. Cancer patients may, therefore, possess abnormal expression of a number of factors which are crucial for normal hemostasis, resulting in a general state of hypercoagulability. In addition to their hemostatic effects, many of these molecules are also involved in other physiological systems, most notably angiogenesis. Many components of the coagulation cascade are also involved in tumor neovascularization, tumor cell growth, and metastasis [21]. This has particular relevance for the anti-neoplastic effects of the various antithrombotics as discussed later.

### Thromboprophylaxis in cancer patients

Several evidence-based guidelines are available that delineate appropriate anticoagulation regimens for VTE treatment, primary and secondary prophylaxis, and long-term anticoagulation in cancer patients [1,16-19]. However, despite the existence of these guidelines and the high-risk of VTE in cancer patients, up to 75% of cancer patients do not receive appropriate prophylaxis [26]. Cancer patients are significantly less likely than non-cancer patients to have received thromboprophylaxis prior to DVT occurrence [27]. In a sub-group analysis of the Epidemiologic International Day for the Evaluation of Patients at Risk for Venous Thromboembolism in the Acute Hospital Care Setting (ENDORSE) [28] worldwide study comprising 1,767 cancer patients undergoing abdominal, gynecological or urological surgery, 27.7% of patients did not receive appropriate thromboprophylaxis [29].

The use of thromboprophylaxis in cancer patients is complicated by the fact that although they are at an increased risk of VTE, they are also at an increased risk of bleeding [30,31]. Given that the risk of VTE outweighs the risk of bleeding in most cancer patients [30,31], the use of antithrombotic agents which provide stable anticoagulation while minimizing bleeding complications is especially important in this high-risk population. In addition, the available guidelines are not always consistent in their recommendations, often give several drugs equally weighted recommendation, and do not always specify the appropriate treatment duration [1,16-19]. Given these ambiguities, it can be difficult for oncologists to make informed decisions about which anticoagulant and treatment regimen would be of most benefit to their patients.

### Choosing the appropriate thromboprophylactic agent

The principal role of the antithrombotics in surgical patients is to provide effective anticoagulation over the course of the increased risk of VTE (during and post-surgery) with the minimum of adverse events such as post-operative bleeding. For cancer patients, there is also increasing evidence that antithrombotics may possess anti-neoplastic effects and are potentially associated with a reduced incidence of cancer and increased survival times when given for long-term prophylaxis [32]. Such findings reinforce the importance of thromboprophylaxis in oncology patients.

When deciding which drug to prescribe, each thromboprophylactic agent can be assessed in terms of three main properties; their efficacy and safety profiles, practicality of use, and cost-effectiveness. The relative merits of the major anticoagulants according to these criteria, as well as any possible anti-neoplastic effects are discussed below.

### Efficacy and safety profiles

#### Primary prophylaxis

A meta-analysis of historical studies performed before thromboprophylaxis was routinely prescribed, demonstrated that low-dose unfractionated heparin (UFH) significantly reduced the risk of combined symptomatic and asymptomatic DVT compared with no prophylaxis (8.7% vs. 25.2%; p < 0.001) in patients undergoing moderate- and high-risk general surgery, without increasing major bleeding (0.33% vs. 0.33%; p = 0.99). In a sub-analysis limited to cancer patients, UFH reduced the incidence of DVT from 30.6% to 13.3% (p < 0.001) [33]. Later meta-analyses comparing UFH and LMWHs have shown that the LMWHs are at least as effective in the primary prophylaxis of VTE after general surgery [34-36] and more effective following major orthopedic surgery [35,37], with comparable or reduced risk of bleeding.

A systematic review of 26 randomized controlled trials comprising 7,639 cancer surgery patients highlighted the importance of post-operative thromboprophylaxis with either UFH or LMWH in these high-risk patients. The overall incidence of combined symptomatic and asymptomatic DVT was reduced from 35.2% in controls to 12.7% in those who received pharmacological prophylaxis [38]. However, few studies have directly compared UFH and LMWH in cancer patients (Table 1) [39-45].

**Table 1:** Summary of VTE and Major Bleeding Rates in Trials Comparing Primary Thromboprophylaxis Strategies in Cancer Patients

**Ref**.	Cancer	Patients, N	Drug	VTE Detection	VTE, %	Bleeding*, %
[39]	Abdominal, thoracic	241	Orgaran 750 IU BID	Asymptomatic VTE: ^125^I-fibrinogen and venography. Symptomatic VTE: DVT - venography; PE - chest X-ray, VPS	10.4	9.0 (overall)
				
		249	UFH 5,000 U BID		14.9	10.6 (overall)

[40]	Abdominal, pelvic	312	Enoxaparin 40 mg SC	Symptomatic and asymptomatic VTE: DVT - venography; PE - VPS, pulmonary angiography	14.7	14.6 (minor) 4.1 (major)
				
		319	UFH 5,000 IU SC TID		18.2	14.3 (minor) 2.9 (major)

[41]	Gynecological	160	Embolex 3,000 IU OD	Asymptomatic VTE: impedance plethysmography, phlebography	6.3	16.9 (wound hematoma)
				
		164	UFH 5,000 IU TID		6.1	28.7 (wound hematoma)

[42]	Gynecological	47	Enoxaparin 2,500 IU OD	Symptomatic VTE: DVT - ultrasonography, venography; PE - VPS, pulmonary anteriography	None	No significant difference (rates not given)
					
		55	UFH 5,000 IU TID		None	

[43]	Colorectal (all patients)^†^	674	Enoxaparin 40 mg SC OD	Symptomatic and asymptomatic VTE: DVT - ultrasonography, venography; PE - VPS, ultrasonography, venography, pulmonary angiography	9.4	10.1 (total) 2.7 (major)
				
		675	UFH 5,000 IU SC TID		9.4	6.2 (total) 1.5 (major)
			
	Cancer sub-group	241	Enoxaparin 40 mg SC OD		13.9	Not reported
					
		234	UFH 5,000 IU SC TID		16.9	

[44]	Abdominal (all patients)^†^	1,425	Dalteparin 5,000 IU OD	Symptomatic and asymptomatic VTE: DVT - ultrasonography, venography; PE - lung scan, pulmonary angiography, helical computed tomography, autopsy	6.1	2.5 (major)
				
		1,433	Fondaparinux 2.5 mg SC OD		4.6	3.4 (major)
			
	Cancer sub-group	712	Dalteparin 5,000 IU OD		7.7	Not reported
					
		696	Fondaparinux 2.5 mg SC OD		4.7	

[45]	Colorectal	486	Enoxaparin 40 mg SC OD	Symptomatic and asymptomatic VTE: DVT - ultrasonography, venography; PE pulmonary angiography, autopsy	12.6	11.5 (major)
				
		464	Nadroparin 2,850 IU OD		15.9	7.3 (major)

BID, twice daily; DVT, deep vein thrombosis; OD, once daily; PE, pulmonary embolism; SC, subcutaneous; TID, three times daily; VPS; ventilation perfusion scan; VTE, venous thromboembolism; UFH, unfractionated heparin.

*For definitions of major bleeding see original studies.

^†^Included non-cancer patients.

The Enoxaparin and Cancer (ENOXACAN) study compared the LMWH enoxaparin and UFH in 1,115 patients undergoing abdominal or pelvic cancer surgery. Enoxaparin (40 mg once daily [OD]) was associated with a significantly lower rate of combined symptomatic and asymptomatic VTE than UFH 5,000 IU three-times daily (14.7% vs. 18.2%, respectively; 95% CI of the difference: -9.2 to -2.3) without increasing the risk of major bleeding (4.1% vs. 2.9%; p = not significant) [40]. A recent systematic review compared the outcomes in cancer patients who received peri-operative thromboprophylaxis with LMWH or UFH, and also found them to be comparable in terms of efficacy (combined symptomatic and asymptomatic DVT: relative risk [RR] 0.73; 95% CI: 0.23-2.28) and safety (major bleeding: RR 0.95; 95% CI: 0.51-1.77). However, in a post-hoc analysis of DVT incidence irrespective of diagnostic modality, LMWH was found to be superior to UFH (DVT: RR 0.72; 95% CI: 0.55-0.94) [46].

The Pentasaccharide General Surgery Study (PEGASUS) trial compared VTE prophylaxis following major abdominal surgery (including cancer surgery) between the synthetic pentasaccharide fondaparinux (2.5 mg started 6 hours after surgery) and the LMWH dalteparin started 2 hours before the operation (first two doses at 2,500 IU, and thereafter 5,000 IU daily). In a sub-group analysis of cancer patients (*n *= 1,408) the combined symptomatic and asymptomatic VTE rate was 4.7% with fondaparinux compared with 7.7% in the dalteparin group (RR reduction [RRR] 38.6%; 95% CI: 6.7-59.6). The rate of major bleeding was not significantly different (3.4% vs. 2.5%, respectively; p = 0.355) [44].

Overall, the available evidence indicates that thromboprophylaxis significantly reduces the risk of VTE in cancer patients undergoing surgery, and that for primary prophylaxis the LMWHs are at least comparable to UFH in terms of efficacy and safety. There are currently limited data directly comparing LMWH with fondaparinux in cancer patients.

#### Extended-duration primary thromboprophylaxis

Increased risk of VTE following major surgery has been shown to extend for several weeks post-operatively [4,47,48]. The prospective, observational @RISTOS project evaluated the incidence and timings of clinically overt VTE across a wide spectrum of patients undergoing cancer surgery. The study reported that the mean time to VTE following cancer surgery was 17.2 days (range, 2-58), with 40% of cases occurring more than 21 days post-operatively. However, these timings exceed the standard duration of prophylaxis which has historically been limited to inpatient therapy. Accordingly, cancer surgery patients receiving only inpatient thromboprophylaxis may be at risk of late VTE events. In the @RISTOS project, 81.7% of cancer surgery patients received in-hospital thromboprophylaxis, whereas only 30.7% continued to receive prophylaxis following discharge [4].

There is considerable evidence to suggest that extended-duration thromboprophylaxis may benefit cancer patients (Table 2) [48-50]. The ENOXACAN II study demonstrated that extended-duration prophylaxis with the LMWH enoxaparin (40 mg OD for 27-31 days) significantly reduced the combined incidence of symptomatic and asymptomatic VTE in cancer patients undergoing abdominal surgery compared with those receiving enoxaparin 40 mg OD for a standard duration of 6-10 days (4.8% vs. 12.0%, respectively; p = 0.02) without increasing bleeding complications (6.1% vs. 4.8%; p = not significant) [48]. The Fragmin After Major Abdominal Surgery (FAME) study of the LMWH dalteparin, which comprised 400 surgical patients of whom approximately half underwent surgery for malignancy, demonstrated that extending the duration of prophylaxis from 1-4 weeks significantly reduced the incidence of VTE without increasing bleeding [49]. In a sub-analysis of cancer patients from this study, extended-duration dalteparin significantly reduced the rate of combined symptomatic and asymptomatic VTE at 4 weeks compared with placebo (8.8% vs. 19.6%, respectively; RRR 55%; p = 0.03) [50]. Taken together, these data confirm that extended-duration prophylaxis reduces VTE in this high-risk population.

**Table 2:** Summary of VTE and Major Bleeding Rates in Trials Comparing Extended-Duration Thromboprophylaxis Strategies in Cancer Patients

**Ref**.	Cancer	Patients, N	Drug	VTE Detection	VTE, %	Bleeding*, %
[48]	Gastrointestinal, genitourinary, gynecological	167	Enoxaparin 40 mg SC 6--10 days plus placebo 19--21 days	Symptomatic and asymptomatic VTE: DVT - venography; PE - VPS, pulmonary angiography	12	3.6 (minor) 3.6 (major)
				
		165	Enoxaparin 40 mg SC 25--31 days		4.8	4.7 (minor) 5.1 (major)

[49]	Abdominal (all patients)^†^	178	Dalteparin 5,000 IU OD plus GCS for 7 days	Symptomatic and asymptomatic VTE: DVT - venography; PE - VPS, spiral computerized tomography, autopsy	16.3	0.9 (minor) 1.8 (major)
				
		165	Dalteparin 5,000 IU SC OD plus GCS for 7 days, plus further 21 days		7.3	1.5 (minor) 0.5 (major)

[50]	Abdominal cancer sub-group	198 total	Dalteparin 5,000 IU OD plus GCS for 7 days		19.6 proximal DVT: 10.4	Not reported
					
			Dalteparin 5,000 IU SC OD plus GCS for 7 days, plus further 21 days		8.8 proximal DVT: 2.2	

DVT, deep vein thrombosis; GCS, graduated compression stockings; OD, once daily; PE, pulmonary embolism; SC, subcutaneous; VPS; ventilation perfusion scan; VTE, venous thromboembolism.

* For definitions of major bleeding see original studies.

^†^Included non-cancer patients

#### Prophylaxis in medical cancer inpatients

There is a growing awareness of the risks of VTE in hospitalized medical patients. Medical patients account for approximately 60% of all hospital admissions and an estimated 50-70% of all symptomatic inpatient VTE events and 70% of all fatal PE events occur in medical, rather than surgical patients [51]. Hospitalization for non-surgical reasons is in itself a risk factor for VTE and the presence of cancer, and its corollary risk factors, further compounds the risk of developing a VTE event [11]. Accordingly, guidelines include recommendations for routine thromboprophylaxis in medical cancer patients [1,16-19]. However, in the Fundamental Research in Oncology and Thrombosis (FRONTLINE) survey only 5% of medical oncologists reported that they routinely used VTE prophylaxis in their cancer patients [52].

Trials investigating thromboprophylaxis in medical patients hospitalized for acute medical illnesses have reported clinical benefits in terms of a significant reduction in VTE without increasing the rates of major bleeding [53-55]. In the Prophylaxis in Medical Patients with Enoxaparin (MEDENOX) study, the use of the LMWH enoxaparin (40 mg subcutaneous [SC] OD) for 6-14 days significantly reduced the incidence of combined symptomatic and asymptomatic VTE compared with placebo (5.5% vs. 14.9%, respectively; RR 0.37; 97.6% CI: 0.22-0.63; p < 0.001). Major bleeding occurred in 1.7% of patients treated with enoxaparin compared with 1.1% in the placebo group [53]. In a sub-group analysis of patients that had active or previously diagnosed cancer, VTE rates were 19.5% (8/41) in the placebo group compared with 9.7% (3/31) in those treated with enoxaparin (RR 0.50; 95% CI: 0.14-1.72; p = 0.4) [56].

Likewise in the Prospective Evaluation of Dalteparin Efficacy for Prevention of VTE in Immobilized Patients Trial (PREVENT), which included 3,706 medical inpatients, of which 190 had cancer, the use of dalteparin (5,000 IU SC OD) for 14 days reduced the combined incidence of symptomatic and asymptomatic VTE from 4.96% in the placebo group to 2.77% (RR 0.55; 95% CI: 0.38-0.80; p = 0.0015). Major bleeding was not significantly increased in the dalteparin group compared with placebo (0.49% vs. 0.16%, respectively; p = 0.15) [54].

In the Arixtra for Thromboembolism Prevention in a Medical Indications Study (ARTEMIS), 849 medical inpatients were randomized to receive fondaparinux (2.5 mg SC OD) or placebo for 6-14 days [55]. In patients with previous or current cancer VTE occurred in 17% (95% CI: 7.6-30.8) of the fondaparinux group compared to 3.9% (95% CI: 0.5-13.5) of the placebo group [57]. However, it should be noted that in all these trials cancer patients represented a sub-group of the total medical patients enrolled and accordingly any conclusions based on these data should reflect the relatively small sample sizes.

Whilst guidelines delineate the appropriate clinical responses for medical inpatients bedridden with cancer or cancer patients hospitalized for a medical illness, they are less clear regarding ambulatory outpatients, and patients receiving highly thrombogenic chemotherapy. For instance, the American College of Chest Physicians (ACCP) guidelines recommend that all cancer patients bedridden with an acute medical illness receive thromboprophylaxis (Grade 1A), but caution against routine thromboprophylaxis in medical patients receiving chemotherapy (Grade 1 C) [1]. Similarly, the American Society of Clinical Oncology (ASCO) guidelines advocate that all hospitalized cancer patients receive thromboprophylaxis, but recommend against routine thromboprophylaxis in ambulant cancer patients receiving chemotherapy [17]. The ASCO guidelines also recommend that ambulant patients receiving thalidomide or lenalidomide with chemotherapy or dexamethasone receive thromboprophylaxis with a VKA targeted to an INR of 1.5 [17]. The Italian Association of Medical Oncology (AIOM) recommend that hospitalized cancer patients confined to bed receive thromboprophylaxis with a LMWH (Level of evidence (LOE) I, Grade A), but do not recommend routine prophylaxis in advanced cancer patients receiving chemotherapy (LOE II, Grade B) or in ambulatory cancer patients receiving adjuvant chemotherapy or hormone therapy (LOE I, Grade A) [16]. In contrast, the National Comprehensive Cancer Network (NCCN) guidelines recommend that patients on highly thrombogenic chemotherapy be considered for thromboprophylaxis, but do not specify the preferred pharmacotherapeutic strategy [19].

Recently, risk assessment models (RAMs) have been developed specifically for VTE risk in cancer patients [58-60]. These models assign cancer patients with a overall VTE risk score by 'adding' thrombogenic risk factors from a variety of patient-, cancer- and treatment-related factors including age, race and immobility status, together with site or stage of the cancer, whether or not the patient is receiving chemotherapy, presence of biomarkers such as platelet count, tissue factor or D-dimer levels [58-60]. These risk scores can aid in determining who is at risk of VTE and warrant thromboprophylaxis.

#### Secondary/long-term prophylaxis

Guidelines recommend that the use of the oral anticoagulant warfarin in cancer patients is limited to long-term secondary prophylaxis following a VTE event or for long-term anticoagulation [18,19]. The guidelines note that cases of VTE should be initially treated with either UFH or a LMWH [18,19], or LMWH [16,17]. In a recent systematic review of the literature, the use of LMWH over UFH for the initial treatment of VTE was associated with significantly reduced mortality rates in cancer patients (RR 0.71; 95% CI: 0.52-0.98) [61]. Data on bleeding were unavailable for this analysis.

As a result of their suitability for outpatient use, the LMWHs can also be considered for use in secondary/long-term prophylaxis, a role traditionally performed by warfarin. In a meta-analysis of studies investigating the efficacy and safety of long-term treatment of VTE, the LMWHs were associated with non-significant reductions in the rates of recurrent symptomatic and asymptomatic VTE (odds ratio [OR] 0.66; 95% CI: 0.41-1.07) and major bleeding (OR 0.45; 95% CI: 0.18-1.11) compared with oral anticoagulants [62]. In cancer patients, treatment of VTE with the LMWH dalteparin was associated with a lower rate of symptomatic, objectively confirmed recurrent thromboembolism compared with warfarin (8.0% vs. 15.8%, respectively; hazard ratio [HR] 0.48; p = 0.002), without increasing the rate of major bleeding (6% vs. 4%) or any bleeding (14% vs. 19%) [63]. Similar findings were reported in a study comparing long-term treatment with the LMWH tinzaparin with warfarin; 7% of patients treated with tinzaparin and 16% of those treated with warfarin experienced symptomatic, objectively confirmed recurrent VTE (RR 0.44; 95% CI: -21.7 to -0.7; p = 0.044) [64]. Bleeding, which was largely minor, occurred in 27% of patients receiving tinzaparin and 24% receiving warfarin (absolute difference -3.0; 95% CI: -9.1 to 15.1).

A recent systemic review concluded that LMWHs for long-term treatment of VTE in patients with cancer reduce VTE compared with warfarin (HR 0.47; 95% CI: 0.32-0.71) [65]. There was no statistically significant difference between the LMWHs and warfarin in bleeding outcomes in this study (RR 0.91; 95% CI: 0.64-1.31). On the strength of these findings the available guidelines increasingly recommend the LMWHs over warfarin for VTE treatment [16-19].

### Practicality of use

The use of UFH is complicated by its narrow therapeutic range, resulting in frequent monitoring of activated partial thromboplastin time or anti-Xa levels, and dose adjustments may be required during treatment [66]. UFH has sub-optimal bioavailability and accordingly when it is given subcutaneously it requires much larger doses than when given intravenously to achieve equivalent anticoagulation levels [67]. The heterogonous assortment of molecules that make up UFH result in a highly polygamous mixture capable of binding multiple plasma proteins, macrophages, and endothelial cells [68]. Accordingly, resulting anticoagulation responses to UFH can vary widely. In contrast, the LMWHs have higher bioavailability [69] and, because of their reduced plasma protein binding, have a more predictable pharmacokinetic profile [70] and a longer half-life [68]. Unlike UFH which is hepatically cleared, the LMWHs are renally excreted. As such, monitoring may be required in certain patient populations, including the morbidly obese, those with severe renal impairment, and pregnant women [68]. However, in the majority of patients, the pharmacokinetic advantages of the LMWHs mean they can be effectively administered without the need to monitor the anticoagulant effect, both in inpatient and outpatient settings.

#### Outpatient use

Although warfarin has historically been the mainstay of long-term thromboprophylaxis, its use is complicated by its narrow therapeutic window and the difficulty in maintaining appropriate levels of anticoagulation [71]. Warfarin is affected by numerous interactions with a wide-range of drugs, nutritional supplements and herbal remedies, and its efficacy can be affected by vomiting and diarrhea, all of which are common in cancer patients. Intolerance, hypersensitivity, and resistance to warfarin leading to treatment failure have all been reported [71]. The effectiveness of warfarin is especially compromised in cancer patients [72]. Compared with matched controls, cancer patients on warfarin spent significantly less time inside the target international normalized ratio (INR) range (both supra- and sub-therapeutic, 54% vs. 64%, respectively; p < 0.001), had more variable INR values (p < 0.001), and had more thrombotic events compared with matched non-cancer patients (p < 0.001) [72]. Warfarin use in cancer patients undergoing chemotherapy results in extra utilization of hospital resources, especially through increased day visits associated with warfarin monitoring and resulting laboratory costs [73]. A further potential limitation of warfarin therapy is that it has a slow onset and long duration of action (half-life 36-42 hours) [74], which can represent a problem if anticoagulation needs to be interrupted quickly for invasive procedures, as is frequently the case in cancer patients.

The principal practical advantage of the LMWHs in the outpatient setting, both for extended-duration primary prophylaxis and long-term secondary prophylaxis, is the lack of a need to monitor anticoagulation in the majority of patients. Patient compliance with LMWHs is high in both non-cancer [75] and cancer populations [76,77], with the majority of patients being comfortable with self-administration. Outpatient thromboprophylaxis in cancer patients with SC LMWH is associated with a good safety profile and a high level of compliance [77]. In a study of VTE treatment in cancer patients, LMWH was associated with improved quality-of-life over warfarin, primarily on the basis of reduced blood tests and increased optimism regarding therapy [78]. Similarly in a pharmacoeconomic analysis using data from the Comparison of Low Molecular Weight Heparin Versus Oral Anticoagulant Therapy for Long Term Anticoagulation in Cancer Patients With Venous Thromboembolism (CLOT) trial which compared the LMWH dalteparin and warfarin for the long-term anticoagulation of cancer patients with DVT, dalteparin was the preferred treatment in 96% (23/24) of respondents and was associated with a gain of 0.157 quality-adjusted life years (QALY) [79].

Overall, therefore, LMWHs offer considerable advantages over warfarin in cancer patients in terms of practicality of use as well as in efficacy.

### Cost comparisons

The costs involved in managing VTE in general are considerable. In the US managing DVT alone is estimated to cost around $1.5 billion annually [80]. The yearly direct costs for treating an individual VTE episode are high; $10,804 for a DVT event and $16,644 for a PE event [81]. Furthermore, the long-term sequelae of VTE such as post-thrombotic syndrome can further increase costs [82]. Accordingly, the expenditure associated with the provision of appropriate anticoagulation following surgery can be offset by the savings achieved by averting the costs of VTE management.

A number of studies have indicated that the LMWHs are economically superior to UFH both for DVT treatment [80,83,84], and for bridging to long-term anticoagulation [85-87], and they are at least non-inferior or superior to warfarin in preventing VTE following orthopedic surgery [88,89]. However, there are limited data regarding the economics associated with thromboprophylaxis following cancer surgery. Data from the CLOT trial on long-term prophylaxis in cancer patients with DVT showed that overall costs were lower with warfarin than dalteparin (CA $2,003 vs. $4,262, respectively; p < 0.001), primarily due to reduced drug-acquisition costs [79]. However, when patient quality of life was also included in the analysis dalteparin therapy was associated with a cost of around CA $13,800 per QALY gained [79], far below the $50,000 cost per QALY considered to be economically acceptable [90].

### Anticoagulants may be associated with increased survival in cancer patients

Three major studies have indicated that the LMWHs may be associated with a survival benefit in cancer patients that could not be directly linked to a reduction in VTE incidence [91-93]. In the Malignancy and Low Molecular Weight-Heparin Therapy (MALT) trial, cancer patients were randomly assigned to 6-weeks of either the LMWH nadroparin (*n *= 148) or placebo (*n *= 154). At 12 months the overall HR for death was 0.75 (95% CI: 0.59-0.96) with a median survival of 8.0 months in the nadroparin group compared with 6.6 months in the placebo group [91]. Similarly, The Fragmin Advanced Malignancy Outcome Study (FAMOUS) compared the LMWH dalteparin given for 1-year with placebo in cancer patients. The Kaplan-Meier survival estimates for 1, 2, and 3 years after randomization were not different between the dalteparin and placebo groups (p = 0.19). However, in an analysis not planned *a priori*, a sub-group of patients who were alive at 17 months, experienced significantly improved survival estimates at 2- and 3-years following randomization with dalteparin versus placebo (78% vs. 55% and 60% vs. 36%, respectively; p = 0.03) with no increase in major bleeding rates [92]. Notably, these effects were observed long after dalteparin was discontinued, suggesting the survival benefit is not dependent on VTE prophylaxis.

In the CLOT trial, over 600 patients with cancer and VTE were randomized to receive 6-months of warfarin or dalteparin therapy. A survival benefit for LMWH over warfarin was observed in patients with non-metastatic cancer, with a 20% mortality rate in the dalteparin group compared with 36% with warfarin (HR 0.50; 95% CI: 0.27-0.95; p = 0.03). However this benefit was not maintained in patients with metastatic cancer (72% vs. 69%, respectively; HR 1.1; 95% CI: 0.87-1.4; p = 0.46) [93].

Although some studies have suggested that warfarin may also improve survival in cancer patients [94,95] and reduce the incidence of cancer [96], a meta-analysis of 11 studies comparing mortality with the LMWHs versus warfarin demonstrated that although the LMWHs increased survival (RR 0.877; 95% CI: 0.789-0.975; p = 0.015) warfarin did not (RR 0.942; 95% CI: 0.854-1.040; p = 0.239) [32]. Furthermore, patients receiving warfarin therapy also had a significant increase in the risk of major bleeding (RR 2.979; 95% CI: 2.134-4.157; p < 0.0001) whereas those receiving LMWH did not (RR 1.678; 95% CI: 0.861-2.269, p = 0.128). In a systematic review of the literature, heparin (UFH or LMWH) was associated with a survival benefit in cancer patients (HR 0.77, 95% CI 0.65-0.91) without significantly increasing the risk of bleeding (RR 1.78, 95% CI 0.73-4.38) [97]. When analyzed by subgroups however, a statistically significant survival benefit was observed in patients with limited small-cell lung cancer (SCLC) (HR 0.56, 95% CI 0.38-0.83), but was not seen in patients with more extensive SCLC (HR 0.80, 95% CI 0.60-1.06) or patients with advanced disease (HR 0.84, 95% CI 0.68-1.03) [97].

Thus it appears that the LMWHs may be associated with improved survival in certain cancer populations. However, more studies are needed to fully characterize this effect and how it is affected by different cancer locations, types, and disease stage. Accordingly, current evidence-based guidelines delineating appropriate thromboprophylaxis and VTE-treatment in cancer patients do not recommend the use of primary thromboprophylaxis to try to improve survival in cancer patients, and use of a LMWH for this indication would be off-label [1,17,19].

Findings suggest that the improvements in survival seen with the LMWHs in cancer patients do not simply result from a decrease in the incidence of VTE, but also from potential anti-neoplastic properties of heparins. Heparin and its derivatives possess mucopolysaccharide chains similar to cell-surface and extra-cellular matrix molecules, raising the possibility that UFH and LMWHs can modulate how cells interact with their environment, enzymes, and cell-signaling molecules, and so affect malignant cell growth [98]. In vivo evidence suggests that the anti-metastatic effects of heparins depend upon P-selectin-mediated binding via their polysaccharide chains rather than their antithrombotic activity [99]. Accordingly fondaparinux, which lacks a polysaccharide chain, did not inhibit metastasis at clinically relevant anticoagulation levels in this model [99]. Similarly, UFH, LMWHs and oligosaccharide truncates of heparin have been shown to inhibit tumor growth and metastasis in vivo [100].

Heparin and oligosaccharide truncates of heparin have also been shown to inhibit angiogenesis [101]. Studies have demonstrated UFH and LMWH have dose-dependent antiangiogenic effects that are mediated via release of endothelial tissue factor pathway inhibitor, which are independent of their antithrombotic activity [102]. Furthermore, heparins can directly affect the immune system by their inhibitory effects on extravasation of leukocytes and the complement system, or by enhancing the susceptibility of cancer cells to immunologic attacks [103]. Consequently, it is likely the proposed anti-neoplastic effects of heparin and the LMWHs are a combination of direct anti-neoplastic, antiangiogenetic, and immunomodulatory effects, as well as indirect effects resulting from their pleiotropic action on the coagulation system.

Each LMWH has a particular structural profile which in turns gives it specific pharmacokinetic and pharmacodynamic properties [104,105]. Structural differences between the LMWHs such as in the molecular weight, molecule length, end-group composition, carboxyl-to-sulfate group ratio and the proportion of anti-Xa binding domains have been shown to affect the biological activity of the resulting molecule [104,105]. It is possible therefore that the LMWHs possess different anti-metastatic properties to one another. However, it is unclear at present to what extent the structural heterogeneity between the LMWHs translates into clinical differences in the drug's anti-metastatic effect. Current research is investigating separating the anticoagulant and anti-metastatic properties of heparin molecules for use in cancer patients [106].

The complex mechanisms associated with improvements in survival of cancer patients treated with heparins are of relevance to the new generation of oral anticoagulants which are under development [107]. In an attempt to separate antithrombotic and bleeding effects, agents have been designed to inhibit specific proteins within the coagulation cascade. However, these new drugs lack the polypharmacological actions of the UFH and LMWHs which are thought to be involved in anti-neoplastic effects, and accordingly it is likely that they will also have concurrent reductions in their anti-neoplastic activity.

## Discussion

Whilst heparin and vitamin K antagonists have been the mainstay of anticoagulant therapy for over fifty years, a new generation of anticoagulants are either recently available or are currently under development, which offer potential benefits over existing therapies in cancer patients [108,109]. For instance, as cancer patients are at an increased risk both of VTE and bleeding [30,31], cancer patients would benefit from anticoagulants that have a favorable balance of anticoagulation to hemorrhagic effects. AVE5026 is a new ultra low-weight LMWH in development whose favorable safety-to-efficacy ratio may make it particularly suitable for a cancer setting [110]. Some of the new anticoagulants, such as dabigatran [111] and rivaroxaban [112,113], are orally administered drugs that have proven effective in reducing the incidence of VTE following major orthopedic surgery. Whilst these new drugs are of potential interest in cancer because they don't require laboratory monitoring and are orally administered, they have yet to be fully tested in an oncology setting.

It is important to remember that the cancer populations discussed in the manuscript frequently comprise heterogeneous patient groups. The mortality rate and risk of VTE in one cancer population may not be equivalent to another population, and likewise the risks and benefits of particular anticoagulation therapies may not necessarily the same either. Furthermore, as patients with advanced cancer, renal or hepatic impairment, or with a short-term prognosis are frequently excluded from clinical trials, the patients included in clinical trials are typically in better health than cancer patients seen in clinical practice.

## Conclusion

Cancer patients are at increased risk of VTE due to a range of disease-, treatment- and patient-related factors. Current evidence-based guidelines support the use of anticoagulation in at-risk surgical cancer patients, although generally there is limited specific guidance as to which anticoagulant is most appropriate, or for how long treatment should be given. Both UFH and the LMWHs are recommended for primary prophylaxis following cancer surgery. Studies show that the LMWHs are at least as effective as UFH in this setting, but are associated with a tendency towards lower bleeding. Given the period of post-operative VTE risk, data increasingly support extended-duration prophylaxis beyond the period of hospitalization. The practical and pharmacokinetic advantages of the LMWHs facilitate such treatment, and the use of LMWHs in this setting is associated with reduced hospital visits and a reduced need for blood tests/monitoring. In other patient populations these advantages mean that the LMWHs are a more cost-effective drug class than UFH, but more specific oncology-based studies are needed before this finding can be applied to cancer patients.

The LMWHs are also recommended for use in secondary/long-term prophylaxis where, compared with warfarin, they display increased efficacy with a good safety profile and reliability, and are associated with increased quality of life. In addition, the LMWHs have been associated with a potential anti-neoplastic effects which may contribute to improved survival times in cancer patients. However, more studies are needed to understand this effect, and the potential role of the LMWHs as antineoplastic therapy.

## Competing interests

The author declares that they have no competing interests.
